# Dual Modulation of Single Molecule Conductance via Tuning Side Chains and Electric Field with Conjugated Molecules Entailing Intramolecular O•••S Interactions

**DOI:** 10.1002/advs.202105667

**Published:** 2022-04-17

**Authors:** Hua Zhang, Wei Xu, Kai Song, Taige Lu, Guanxin Zhang, Yaping Zang, Wenjing Hong, Deqing Zhang

**Affiliations:** ^1^ Beijing National Laboratory for Molecular Sciences Organic Solids Laboratory Institute of Chemistry Chinese Academy of Sciences Beijing 100190 China; ^2^ School of Chemical Sciences University of Chinese Academy of Sciences Beijing 100049 China; ^3^ State Key Laboratory of Physical Chemistry of Solid Surfaces College of Chemistry and Chemical Engineering Xiamen University Xiamen 361005 China

**Keywords:** electric fields, intramolecular conformational tuning, molecular switch, single‐molecule conductance

## Abstract

Herein, single‐molecule conductance studies of **TBT1‐TBT6** which entails 1,4‐dithienylbenzene as the backbone and —SMe groups as the anchoring units, with the scanning tunneling microscope break junction (STM‐BJ) technique, are reported. The molecular conductance of **TBT1** with intramolecular O•••S noncovalent interactions is enhanced by about one order of magnitude in comparison to their analogue **TBT2** (which contains alkyl instead of alkoxy chains). By replacing the methoxy groups in **TBT1** with extending alkoxy chains in **TBT3**, **TBT4**, and **TBT5**, the molecular backbones become twisted and as a consequence the single‐molecule conductance decreases gradually, showing that the intramolecular O•••S noncovalent interaction is influenced by the structural features of alkoxy chains. More importantly, the single‐molecule conductance of **TBT3**, **TBT4**, and **TBT5** can be boosted by increasing the electric field applied to the molecular junctions. Remarkably, the conductance of **TBT3**, **TBT4**, and **TBT5** can be reversibly modulated due to the conformational changes between twisted and planar ones by varying the electric field. These results demonstrate that molecules with intramolecular O•••S noncovalent interactions have the potential for in situ control of the electrical properties of molecular‐scale devices.

## Graphical abstract

Here, single‐molecule conductance studies for molecules with 1,4‐dithienylbenzene as the backbone and —SMe groups as the anchoring units by using the STM‐BJ technique are reported. The results show that the single‐molecule conductance can be tuned by varying side chains and electric field through influencing the intramolecular O•••S interactions and molecular conformation within the metal–molecule–metal junctions.

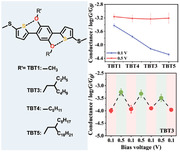

## 1 Introduction

Single‐molecule break junction techniques,^[^ ^1^ ^]^ such as STM‐BJ^[^ ^2^ ^]^ and mechanically controllable break junction,^[^ ^3^ ^]^ have been used to explore the relationship between conductance and structures of molecular wires and construct responsive single‐molecule devices in the past years.^[^ ^4^ ^]^ Interestingly, conductance of molecular junctions linked by intermolecular hydrogen bonding, *π*‐*π* stacking, coordination interaction and host‐guest interaction was successfully measured and investigated.^[^ ^5^ ^]^ However, investigations of intramolecular noncovalent interactions through single molecular junction were less conducted.^[^ ^5d^ ^]^

Recently, intramolecular O•••S noncovalent interaction have been proposed for designing planar conjugated molecules for high performance organic opto‐electric materials.^[^ ^6^, ^7^ ^]^ Although intramolecular O•••S noncovalent interaction was proved by analysis including single crystal X‐ray diffraction, ultraviolet–visible absorption spectroscopy, nuclear magnetic resonance spectra and density functional theory (DFT) computation,^[^ ^7^, ^8^ ^]^ there is still a lack of method by means of the single‐molecule junction. It is significant to explore the effects of structural parameters and external stimuli such as electric field on intramolecular O•••S noncovalent interaction. In addition, molecules with noncovalent conformational interactions have the advantage for constructing molecular devices with responsiveness and reversibility.^[^ ^9^, ^10^ ^]^ More importantly, the strong electric fields within the two nanoelectrodes of the break junction technique offer the unique tool to realize high‐performance responsive molecular devices.^[^ ^11^ ^]^ Thus, it is highly interesting to fabricate molecular level devices by varying the electric fields applied across the junction of molecules with intramolecular noncovalent interactions.

**Figure 1:**
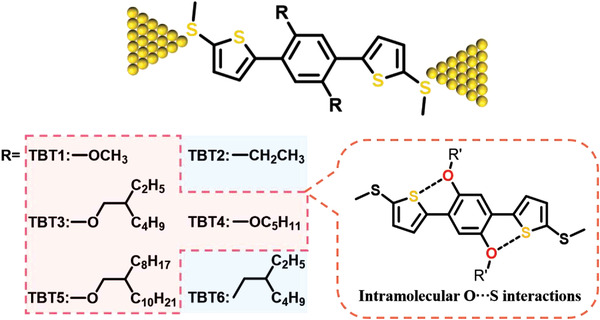
Schematic illustration of single‐molecule junction and molecular structures of **TBT1‐TBT6**.

In this paper, we report the single‐molecule conductance studies of **TBT1‐TBT6** (see **Figure** **1**), which entails 1,4‐dithienylbenzene as the backbone and —SMe groups as the anchoring units, with the STM‐BJ technique. The results show that the molecular conductance of **TBT1** with two methoxy groups is about one order of magnitude higher than that of its analogue **TBT2** (which contains two ethyl groups instead of alkoxy chains). This is attributed to the more planar backbone of **TBT1** induced by the intramolecular O•••S noncovalent interaction. Interestingly, by replacing the methoxy groups in **TBT1** with extending alkoxy chains as in **TBT3**, **TBT4**, and **TBT5**, the single‐molecule conductance decreases gradually under 0.1 V bias voltage. This manifests that the intramolecular O•••S noncovalent interaction are influenced by the structural features of alkoxy groups in **TBT3**, **TBT4**, and **TBT5**. Remarkably, the single‐molecule conductance of **TBT3**, **TBT4**, and **TBT5** can be boosted by increasing the electric field applied to the molecular junctions. It is worth noting that the conductance of **TBT3**, **TBT4**, and **TBT5** can be reversibly modulated due to the conformational changes between twisted and planar ones by varying the electric field. We further rationalize these results through characterizations including single crystal X‐ray diffraction, ultraviolet–visible absorption spectroscopy, nuclear magnetic resonance spectra and DFT computations. Our work demonstrates for the first time that molecular conformation can be regulated through intramolecular O•••S noncovalent interactions in single molecular electronics via an external field, and molecules with intramolecular O•••S noncovalent interaction have the potential to be used for single‐molecule electrical switches.

## 2 Results and Discussion

**Figure 2:**
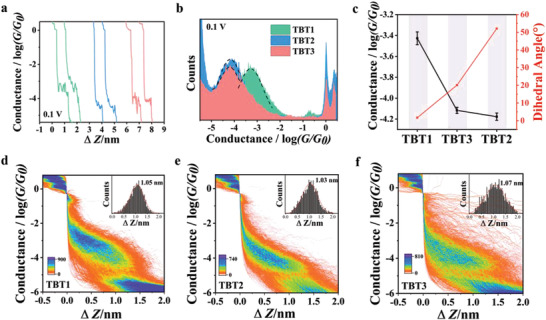
a) Typical individual traces of **TBT1** (green), **TBT2** (blue), and **TBT3** (orange) under 0.1 V bias voltage. b) 1D conductance histograms of **TBT1** (green), **TBT2** (blue), and **TBT3** (orange) under 0.1 V bias voltage. c) Comparison of molecular conductances of **TBT1**‐**TBT3** and dihedral angles of **TBT1**‐**TBT3**. d–f) 2D conductance histograms of **TBT1**, **TBT2**, and **TBT3** under 0.1 V bias voltage. The distance distributions are shown in the insets.

To verify the intramolecular O•••S noncovalent interactions, we synthesized three molecules **TBT1‐TBT3** and investigated the single‐molecule charge transport properties by conductance measurement using STM‐BJ methods. Their molecular structures are shown in Figure 1, and the alkyl and alkoxy chains were introduced into the benzene of the conjugated backbone flanked by —SMe anchors. The individual stretching traces of conductance‐displacement for **TBT1‐TBT3** in **Figure** **2**a indicated the formation of single‐molecule junctions and the conductance changes were clearly observed. As shown in 1D conductance histograms (Figure 2b), **TBT1** with strong O•••S noncovalent interactions shows the highest Gaussian fitted conductance peak around ≈10^−3.30^ G_0_ at 0.1 V, while the conductance of **TBT2** (≈10^−4.19^ G_0_) without O•••S noncovalent interactions is around one order of magnitude lower than **TBT1**. **TBT3** is the reference compound with a large alkoxy groups (2‐ethyl‐hexyl group) on the oxygen atom. The conductance value of **TBT3** (≈10^−4.10^ G_0_) was almost equal with that of **TBT2**, indicating the interaction of O•••S intensity was weakened by the introduced bulky group. The 2D conductance histograms were constructed by at least 2000 individual traces, as shown in Figure 2d–f. A clear conductance plateau can be observed for each 2D conductance histogram. Further Gaussian analysis of the plateau length distribution in each 2D conductance histogram revealed that the stretching distances of **TBT1‐TBT3** were all close to 1.05 nm as shown in the insets of Figure 2d–f. After correcting by a 0.5 nm snap‐back distance of the gold‐gold atomic contact breaking, the calibrated stretching distance for **TBT1‐TBT3** should be around 1.55 nm. This value is consistent with the distance between the theoretical distance of the two —SMe anchoring groups (1.4 nm) in molecular backbones, suggesting that these junctions are indeed formed across the molecular backbone. We hence hypothesize that the high conductance observed in **TBT1** arises from the intramolecular O•••S noncovalent interactions effect.

**Figure 3:**
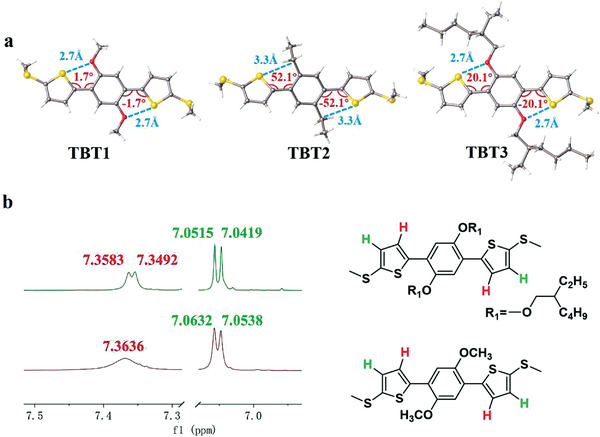
a) Top views of the single crystal structures of **TBT1**, **TBT2**, and **TBT3**. b) ^1^H NMR spectra for **TBT1** and **TBT3** in CDCl_3_.

To further support the hypothesis that the intramolecular O•••S noncovalent interactions induced conductance enhancement, the precise configurations of molecules **TBT1‐TBT3** were obtained by single crystal X‐ray diffraction (**Figure** **3**a) and ^1^HNMR analysis (Figure 3b and Figure S14, Supporting Information). The crystal structure of **TBT1** shows that the distances between alkoxy oxygen and thiophene sulfur atoms (2.707(3) Å) are remarkably shorter than the sum of the van der Waals radii of the two atoms (3.25 Å) and such strong intramolecular O•••S noncovalent interaction induces the *π*‐conjugated block to be a highly planar conformation with a small torsion angle of 1.7°. In contrast, the crystal structure of **TBT2** displays a twisted configuration with 52.1° dihedral angle between adjacent thiophene and benzene rings. For molecule **TBT3**, structural analysis reveals that the distance between oxygen and sulfur is 2.742 (2) Å, which is slightly longer than that in **TBT1**. However, the existence of alkoxy chains leads to a 20.1° dihedral angle with the molecular backbones. Although the dihedral angle is smaller than **TBT2**, it is not surprising to predict that the flexible alkoxy chain will result in an increasing torsion angle in the solution. Figure 2c shows that the dihedral angle between adjacent thiophene and benzene rings follows the trend of **TBT1 < TBT3 < TBT2** and the trend of the molecular conductance variation is opposite. It also suggested that the contribution of conductance enhancement is mainly dominated by the O•••S noncovalent interaction induced coplanarity rather than the charge transport through the O•••S interaction itself. Intramolecular O•••S noncovalent interactions in **TBT1 and TBT3** were also identified in the ^1^H NMR measured under the same condition. As shown in Figure 3b, hydrogens on thiophenes close to benzene ring were defined as *α*‐H marked in red, the other hydrogens on thiophenes were defined as *β*‐H marked in green**. TBT1** shows two signals for hydrogens on thiophenes, a broad singlet at *δ* 7.3636 for *α*‐H, and a doublet at *δ* 7.0632 for *β*‐H consistent with the structure.^[^ ^12^ ^]^ **TBT3** (*δ* 7.3583 and 7.0515) show two doublets of hydrogens on thiophenes. In contrast, **TBT2** shows two high‐field signals in the ^1^H NMR spectrum at *δ* 7.0612 and 6.9039 (see Figure S14, Supporting Information). As a result, ^1^H NMR signals for both *α*‐H and *β*‐H on thiophenes of **TBT1** and **TBT3** with intramolecular O•••S noncovalent interaction were down‐filed shifted slightly, being consistent with more planar molecular backbone. The above results are consistent with the conductance trend in Figure 2a and support the proposed hypothesis. Additionally, the solution ultraviolet–visible absorption spectroscopy shows that the absorption maxima of **TBT1** (389 nm) are redshifted compared with **TBT2** (319 nm), as shown in Figure S13 (Supporting Information), being consistent with intramolecular noncovalent interactions and planarization in solution. Consequently, the single‐molecule conductance increased with the enhancement of intramolecular O•••S interactions, providing a promising strategy to achieve extended *π*‐electron systems with high charge transport capability.

**Figure 4:**
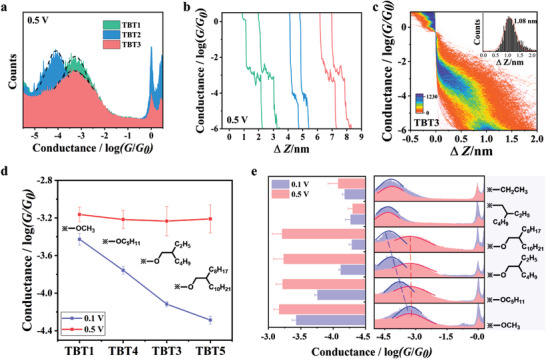
a) 1D conductance histograms of molecule **TBT1** (green), **TBT2** (blue), and **TBT3** (orange) under 0.5 V bias voltage. b) Typical individual traces of **TBT1** (green), **TBT2** (blue), and **TBT3** (orange) under 0.5 V bias voltage. c) 2D conductance histograms of molecule **TBT3** under 0.5 V bias voltage. The distance distributions are shown in the inset. d) Comparison of molecular conductance of molecules with different lengths of alkoxy chains under 0.1 V and 0.5 V bias voltages. e) 1D conductance histograms of molecule **TBT1‐TBT6** under 0.1 V (purple) and 0.5 V (orange) bias voltage.

To clarify intramolecular O•••S noncovalent interactions at high bias, we carried out the conductance measurements at 0.5 V. As shown in **Figure** **4**a, unlike the conductance of **TBT1** with methoxyl groups, the conductance of **TBT3** increased remarkably from ≈10^−4.10^ G_0_ to ≈10^−3.17^ G_0_ when changing the applied bias voltage from 0.1 to 0.5 V. By contrast, there is no apparent change in the conductance of the molecule with ethyl chains at different bias voltages. Furthermore, 2D conductance histograms were constructed to acquire more information from the stretching process (see Figure 4c and Figure S18, Supporting Information). We note from the 2D histograms that the molecular length of **TBT1‐TBT3** all remained unchanged under different applied bias voltages (0.1 V and 0.5 V). As shown in the insets of Figure 4c and Figure S18 (Supporting Information), the measured molecular lengths are in accordance with the calculated ones (≈1.4 nm). These results demonstrate that the variation of molecular conductance observed in different bias voltages is due to the changes of molecular electronic structures instead of altering the electrode‐molecule contact locations. These results show that molecules with intramolecular O•••S noncovalent interactions enable conductance modulations by varying bias between two electrodes. Notably, these conductance modulations are more prominent for molecules with large alkoxy groups.

To further explore the impact of an electric field on the conductance of molecules with intramolecular O•••S noncovalent interactions, we synthesized the other three molecules **TBT4‐TBT6** (see Figure 1) and performed conductance measurements at both 0.1 V and 0.5 V. As shown in Figure 4d,e, the conductance of **TBT4** and **TBT5** increases at 0.5 V. Specifically, the conductance of **TBT4** with *n*‐pentyloxy group increases from ≈10^−3.77^ G_0_ to ≈10^−3.30^ G_0_ by varying the bias from 0.1 to 0.5 V, while **TBT5** with bulky alkoxy groups shows a more obvious conductance increase from ≈10^−4.24^ G_0_ to ≈10^−3.31^ G_0_. By contrast, there is no noticeable conductance change for **TBT6** containing alkyl substitution under 0.1 V (≈10^−4.31^ G_0_) and 0.5 V (≈10^−4.38^ G_0_). The conductance modulation trend for these molecules further implies that the electric field affects the intramolecular O•••S noncovalent interactions and thus the molecular conductance. We therefore hypothesize that the twisted molecules containing large alkoxy groups become more planar under a high electric field, thus yielding a high conductance comparable with that of **TBT1** with more planar conformation.

**Figure 5:**
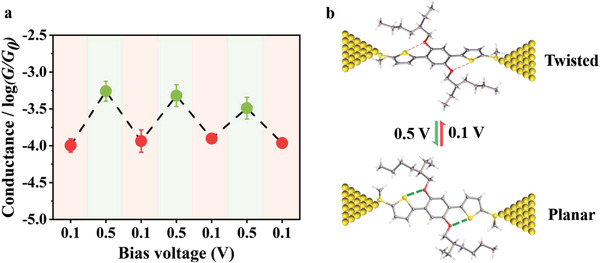
a) Reversible switching cycles of conductance for **TBT3** by varying the bias voltage between 0.1 V and 0.5 V. b) Illustration of conformation change for **TBT3** in single molecular junction by varying the bias voltage between 0.1 V and 0.5 V.

To better explore the conductance modulation of **TBT3** under different bias voltages, we examined the conductance by applying 0.1 V and 0.5 V alternatively using the STM‐BJ technique (see **Figure** **5** and Figure S19, Supporting Information). It can be seen that the conductance of **TBT3** switches reversibly between the high and low conductance states, and the two states show approximately one order of magnitude difference in conductance. These results demonstrated that molecules with intramolecular O•••S noncovalent interactions have the potential for in situ control of electrical switches.

**Figure 6:**
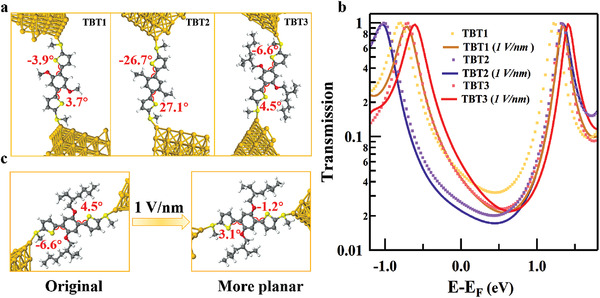
a) The optimized configurations of **TBT1‐TBT3** (from *left* to *right*). b) The transmission spectra of **TBT1‐TBT3** under 0 V nm^−1^ (dot line) and 1 Vnm^−1^ (solid line) electric fields. c) The optimized configurations of **TBT3** under 0 and 1 Vnm^−1^ electric fields.

To further explore the impact of intramolecular O•••S noncovalent interactions on the charge transport in single molecular junctions, we performed density functional theory (DFT) calculations on the junctions formed through Au‐S contacts. As shown in **Figure** **6**a, we first obtained the optimized geometries without application of external field for **TBT1‐TBT3**. In agreement with the above experimental results, the planar conformational molecule **TBT1** shows more planar structure (dihedral angles: −3.9° and 3.7°) than **TBT2** (dihedral angles: −26.7° and 27.1°). For **TBT3**, the dihedral angles (‐6.6°and 4.5°) are also smaller than **TBT2** but larger than **TBT1** due to the steric effect caused by the bulky alkoxy groups. Additionally, we calculated the transmission functions using the nonequilibrium Green's function (NEGF) formalism to further understand the charge transport. Figure 6b shows that the transmission probability at Fermi follows the trend of **TBT1 > TBT3 > TBT2**, which is consistent with the experimental results.

To further explore the effect of the electric field, we performed similar calculations under an applied electric field of 1 Vnm^−1^ (Figure S16, Supporting Information and Figure 6b). Notably, under the electric field, **TBT3** becomes more planar (dihedral angles: 3.1° and −1.2°), while the conformations of **TBT1** and **TBT2** do not show obvious changes. This conformational planarization under a high bias is attributed to the existence of a relatively larger dipole in **TBT3**, and is responsible for the observed conductance increases under a high applied bias voltage (as reflected by the increase of transmissions). The calculation qualitatively explains the conductance enhancement for **TBT3** with intramolecular O•••S noncovalent interaction induced under high electric field.

## 3 Conclusion

In conclusion, we show the enhancement of single‐molecule conductance for molecules with intramolecular O•••S noncovalent interactions. Furthermore, the results reveal that the intramolecular O•••S interactions are influenced by the structural features of alkoxy groups in **TBT3**, **TBT4**, and **TBT5** and the electric field applied to the molecular junctions. Interestingly, it was found that molecules with intramolecular O•••S noncovalent interactions enable conductance modulation by varying the applied electric field between two electrodes, especially those with large alkoxy groups. In particular, the single‐molecule conductance of **TBT3** can be modulated reversibly by one order of magnitude. It is noted that the regulation of molecular conformation through intramolecular O•••S noncovalent interactions in single molecular electronics via external field was never reported before. Consequently, molecules with intramolecular O•••S noncovalent interactions offer an efficient and in situ approach for establishing single‐molecule electrical switches.

## 4 Experimental Section

### Materials

The reagents and starting materials were commercially available and used directly without further purification unless otherwise specified. Gold wires (99.99%, 0.25 nm diameter) were purchased from Beijing Jiaming Platinum Nonferrous Metal Co. Ltd. Crystallographic data (excluding structure factors) reported in this paper were deposited in the Cambridge Crystallographic Data Centre (CCDC No. 2070566 for compound **TBT1**, CCDC No. 2070565 for compound **TBT2**, CCDC No. 2103035 for compound **TBT3**).

### General Synthetic Procedures for **TBT1, TBT3‐TBT5**

To a Schlenk tube equipped with a magnetic stir bar was charged with 1, 4‐dibromo‐2,5‐dialkoxybenzene (1.0 eq.), 5‐(methylthio)thiophene‐2‐boronic acid pinacol ester (3.0 eq.), [Pd_2_(dba)_3_] (0.1 eq.), K_3_PO_4_ (3.0 eq.), SPhos (0.2 eq.) and toluene. The mixture was stirred at 110 °C for 10 h under N_2_ atmosphere. Then the reaction mixture was concentrated under vacuum and the residue was purified by silica gel column chromatography with hexane and CH_2_Cl_2_ as eluent.

### Synthesis of **TBT1**

**TBT1** (221 mg) was obtained as a yellow solid in 33% yield. For compound **TBT1**, m.p. 112.2–112.8 °C. ^1^H NMR (300 MHz, CDCl_3_): *δ* (ppm) 7.37 (s, 2H), 7.18 (s, 2H), 7.07 (s, 2H), 3.95 (s, 6H), 2.54 (s, 6H). ^13^C NMR (75 MHz, CDCl_3_): *δ*(ppm) 149.85, 140.97, 137.36, 130.73, 125.45, 122.75, 111.41, 56.38, 22.05. HR‐MS (MALDI‐TOF): calcd for C_18_H_18_O_2_S_4_ (M^+^) 394.0184; found: 394.0185. Calcd for C_18_H_18_O_2_S_4_: C, 54.79; H, 4.60; S, 32.50; found: C, 54.80; H, 4.63; S, 31.63.

### Synthesis of **TBT3**

**TBT3** (97 mg) was obtained as an yellow solid in 27% yield. For compound **TBT3**, m.p. 35.1–35.7 °C. ^1^H NMR (300 MHz, CDCl_3_): *δ* (ppm) 7.37(d, *J* = 3.0 Hz, 2H), 7.18 (s, 2H), 7.06 (d, *J* = 3.0 Hz, 2H), 3.97 (d, *J* = 3.0 Hz, 4H), 2.53 (s, 6H), 1.89–1.81 (m, 2H), 1.65–1.43 (m, 8H), 1.37–1.31 (m, 8H), 0.98–0.89 (m, 12H).^13^C NMR (100MHz, CDCl_3_): *δ* (ppm) 149.25, 141.31, 137.07, 130.50, 125.30, 122.65, 111.86, 71.91, 39.61, 30.68, 29.15, 24.03, 23.05, 21.99, 14.08, 11.20. HR‐MS(MALDI‐TOF): calcd for C_32_H_46_O_2_S_4_ (M^+^) 590.2375; found: 590.2369. Calcd For C_32_H_46_O_2_S_4_: C, 65.04; H, 7.85; S, 21.70; found: C, 65.13; H, 7.87; S, 21.41.

### Synthesis of **TBT4**

**TBT4** (198 mg) was obtained as an yellow solid in 32% yield. For compound **TBT4**, m.p. 80.0––80.5 °C. ^1^H NMR (400 MHz, CDCl_3_): *δ* (ppm) 7.38 (d, *J* = 4.0 Hz, 2H), 7.17 (s, 2H), 7.06 (d, *J* = 4.0 Hz, 2H), 4.07 (t, *J* = 8.0 Hz, 4H), 2.53 (s, 6H), 1.95–1.88 (m, 4H), 1.54–1.48 (m, 4H), 1.46–1.37 (m, 4H), 0.95 (t, *J* = 8.0 Hz, 6H).^13^C NMR (75MHz, CDCl_3_): *δ* (ppm) 149.14, 141.24, 137.12, 130.57, 125.21, 122.75, 111.98, 69.72, 29.04, 28.38, 22.46, 22.03, 14.04. HR‐MS(MALDI‐TOF): calcd for C_26_H_34_O_2_S_4_ (M^+^) 506.1436; found: 506.1433. Calcd For C_26_H_34_O_2_S_4_: C, 61.62; H, 6.76; S, 25.30; found: C, 61.62; H, 6.74; S, 25.04.

### Synthesis of **TBT5**

**TBT5** (396 mg) was obtained as an yellow liquid in 35% yield. ^1^H NMR (400 MHz, CDCl_3_): *δ* (ppm) 7.36(d, *J* = 4.0 Hz, 2H), 7.17 (s, 2H), 7.04 (d, *J* = 4.0 Hz, 2H), 3.95 (d, *J* = 4.0 Hz, 4H), 2.51 (s, 6H), 1.92–1.86 (m, 2H), 1.59–1.51 (m, 4H), 1.47–1.25 (m, 60H), 0.89 (t, *J* = 8.0Hz, 12H).^13^C NMR (100MHz, CDCl_3_): *δ* (ppm) 149.20, 141.28, 137.04, 130.42, 125.21, 122.60, 111.76, 72.24, 38.14, 31.90, 31.50, 30.04, 29.68, 29.64, 29.61, 29.34, 26.93, 22.67, 21.93, 14.09. HR‐MS(MALDI‐TOF): calcd for C_56_H_94_O_2_S_4_ (M^+^) 926.6131; found: 926.6132. Calcd For C_56_H_94_O_2_S_4_: C, 72.51; H, 10.21; S, 13.82; found: C, 72.72; H, 10.21; S, 13.60.

### General Synthetic Procedures for **TBT2 and TBT6**

To a Schlenk tube equipped with a magnetic stir bar was charged with 1, 4‐dibromo‐2,5‐dialkylbenzene (1.0 eq.), 5‐(methylthio)thiophene‐2‐boronic acid pinacol ester (3.0 eq.), [Pd_2_(dba)_3_] (0.1 eq.), K_3_PO_4_ (3.0 eq.), SPhos (0.2 eq.) and toluene. The mixture was stirred at 110°C for 10 h under N_2_ atmosphere. Then the reaction mixture was concentrated under vacuum and the residue was purified by silica gel column chromatography with hexane and CH_2_Cl_2_ as eluent.

### Synthesis of **TBT2**

**TBT2** (113 mg) was obtained as an yellow solid in 28% yield. For compound **TBT2**, m.p. 71.2–72.1 °C. ^1^H NMR (300 MHz, CDCl_3_): *δ* (ppm) 7.27 (s, 2H), 7.08 (d, *J* = 6.0 Hz, 2H), 6.92 (d, *J* = 3.0 Hz, 2H), 2.80 (dd, *J* = 6.0, 9.0Hz, 4H), 2.55 (s, 6H), 1.20 (t, *J* = 9.0 Hz, 6H).^13^C NMR (75 MHz, CDCl_3_): *δ*(ppm) 144.99, 139.68, 136.87, 133.31, 131.31, 131.12, 126.71, 26.05, 22.17, 15.65. HR‐MS(MALDI‐TOF): calcd for C_20_H_22_S_4_ (M^+^) 390.0599; found: 390.0599. Calcd for C_20_H_22_S_4_: C, 61.49; H, 5.68; S, 32.83; found: C, 61.19; H, 5.69; S, 33.11.

### Synthesis of **TBT6**

**TBT6** (179 mg) was obtained as a yellow liquid in 29% yield. ^1^H NMR (400 MHz, CDCl_3_): *δ* (ppm) 7.17 (s, 2H), 7.06 (d, *J* = 4.0 Hz, 2H), 6.86 (d, *J* = 4.0 Hz, 2H), 2.66 (d, *J* = 8.0 Hz, 4H), 2.53 (s, 6H), 1.47–1.37 (m, 2H), 1.25–1.13 (m, 16H), 0.81 (t, *J* = 8.0 Hz, 6H), 0.74 (t, *J* = 12.0 Hz, 6H).^13^C NMR(100 MHz, CDCl_3_): *δ* (ppm) 145.52, 137.56, 136.71, 133.61, 133.15, 131.21, 126.83, 40.11, 37.25, 32.45, 28.67, 25.67, 22.94, 22.32, 14.05, 10.73. HR‐MS (MALDI‐TOF): calcd for C_32_H_46_S_4_ (M^+^) 558.2477; found: 558.2477. Calcd For C_32_H_46_S_4_: C, 68.76; H, 8.30; S, 22.94; found: C, 68.81; H, 8.26; S, 23.03.

### Single‐Molecule Conductance Measurement

Single‐molecule conductance was measured with the homemade STM‐BJ setup.^[^ ^2a^ ^]^ In brief, a stepper motor and a piezo stack were used to control the distance between the gold tip and substrate. By repeating forming and breaking Au point contacts, molecular junctions were formed. All measurements were carried out in 0.1 x 10^−3^ m solutions of the molecules in 1,2,4‐trichlorobenzene (TCB) at room temperature.

### DFT Calculations

DFT calculations were carried out using the Perdew–Burke–Ernzerhof (PBE) exchange‐correlation functional implemented by the Fritz Haber Institute ab initio molecular simulation (FHI‐aims) packages.^[^ ^13^ ^]^ The geometries of conjugated molecules were optimized first to obtain optimal molecular configurations. The single Au atoms were attached to the S atoms at SMe anchors of the molecules to optimize the geometries further. The Au pyramids with 60 atoms were attached to S atoms, replacing the Au atoms for calculating the transmission across the junctions using the nonequilibrium Green's function (NEGF) formalism. To model the molecular configurations under an electric field, an electric field of 1 V nm^−1^ was applied first to optimize the geometries. After attaching single Au atoms to the two sides of the optimized molecules, the molecular geometries were relaxed under the electric field. After geometry optimization, the Au pyramids with 60 atoms were attached to S atoms replacing the Au atoms for calculating the transmission across the junctions.

### Statistical Analysis

The data analysis was carried out based on the previously reported methods.^[^ ^1^, ^2^, ^10^, ^14^ ^]^ For statistical analysis, thousands of conductance traces were collected to construct logarithmically binned conductance histograms. These conductance histograms show peaks at the quantum of conductance G_0_ (2*e* ^2^/*h*), corresponding to gold−gold atomic contact. After the rupture of Au point‐contacts, molecular conductance below 1 G_0_ showed the formation of molecular junctions. The junction length was calibrated by the direct tunneling distance distribution in pure TCB and the final displacements were obtained by calibrating the snap‐back distance (0.5±0.1 nm). In Figures 2c and 4d,e, each conductance data point was acquired from an average of three independent conductance measurements. In Figures 2b and 4a,e, the conductance histograms were fitted by Gaussian function (*y* = *y*_0_ + *A*/(w*sqrt(*π*/(4*ln(2)))) * exp(−4*ln(2)*(x − x_c_)^2/*w*^2)), which were analyzed with the Goodness of fit (*R* ^2^) and F‐test, significance was defined as *p* < 0.05. The statistical analyses were performed using Origin 9.0.

## Conflict of Interest

The authors declare no conflict of interest.

## Supporting information

Supporting Information

## Data Availability

The data that support the findings of this study are available in the supplementary material of this article.
