# Supplementary material for: Dual Modulation of Single Molecule Conductance via Tuning Side Chains and Electric Field with Conjugated Molecules Entailing Intramolecular O•••S Interactions
Source: Adv Sci (Weinh). 2022 Apr 17;9(17):2105667. doi: 10.1002/advs.202105667 (PMC9189668; doi:10.1002/advs.202105667)
Supplement: Supplementary file 1 — Supporting Information [file ADVS-9-2105667-s001.pdf]

ADVANCED SCIENCE
Open Access

Supporting Information

for *Adv. Sci.*, DOI 10.1002/advs.202105667

Dual Modulation of Single Molecule Conductance via Tuning Side Chains and Electric Field with Conjugated Molecules Entailing Intramolecular O•••S Interactions

Hua Zhang, Wei Xu, Kai Song, Taige Lu, Guanxin Zhang, Yaping Zang, Wenjing Hong and Deqing Zhang**

Supplementary Materials for

Dual Modulation of Single Molecule Conductance via Tuning Side Chains and Electric Field with Conjugated Molecules Entailing Intramolecular O···S Interactions

Hua Zhang, Wei Xu, Kai Song, Taige Lu, Guanxin Zhang, Yaping Zang, Wenjing Hong, and Deqing Zhang**

H. Zhang, K. Song, Prof. G. Zhang, Prof. Y. Zang, Prof. D. Zhang

Beijing National Laboratory for Molecular Sciences, Organic Solids Laboratory, Institute of Chemistry, Chinese Academy of Sciences, Beijing 100190, China.

E-mail: dqzhang@iccas.ac.cn

H. Zhang, Prof. G. Zhang, Prof. Y. Zang, Prof. D. Zhang

School of Chemical Sciences, University of Chinese Academy of Sciences, Beijing 100049, China.

W. Xu, T. Lu, Prof. W. Hong

State Key Laboratory of Physical Chemistry of Solid Surfaces, College of Chemistry and Chemical Engineering, Xiamen University, Xiamen 361005, China.

E-mail: whong@xmu.edu.cn

Table of Contents

1. Materials and characterization techniques........2
2. Single crystal X-ray diffraction analysis........3
3. ^{1}H and ^{13}C NMR spectra........6
4. Absorption spectra of **TBT1**, **TBT2** and **TBT3**........12
5. Comparison of ^{1}H NMR spectra of **TBT1**, **TBT2** and **TBT3**........12
6. Theoretical modelling........14
7. Data analysis of single-molecule conductance........15

1. Materials and characterization techniques

The reagents and starting materials were commercially available and used directly without further purification unless otherwise specified. Toluene was freshly distilled with Na before using. 1,4-dibromo-2,5-dipentyloxybenzene, 1,4-dibromo-2,5-bis(2-ethyl)hexyloxybenzene and 1,4-dibromo-2,5-bis(2-octyl)dodecyloxybenzene were purchased from WO ER JI MING technological development research institute CO., Beijing, China. Gold wires (99.99%, 0.25 nm diameter) were purchased from Beijing Jiaming Platinum Nonferrous Metal Co. Ltd.

^{1}H NMR and ^{13}C NMR spectra were measured with Fourier 300 MHz and Bruker AVANCE III 400 MHz. Mass spectra were collected on a Bruker Solarix-XR high-resolution mass spectrometer. Elemental analyses were performed on a Carlo-Erba-1106 instrument. Melting points were measured on a BÜCHI melting point B-540. Absorption spectra of solutions were recorded on HITACHI UH4150 UV−Vis spectrophotometer. Single crystals used for X-ray diffraction analysis were obtained by slowly diffusing methanol into the toluene solutions. The data for the single crystals of **TBT1**, **TBT2** and **TBT3** were collected with a Rigaku Saturn diffractometer with CCD area detector.

2. Single crystal X-ray diffraction analysis

Table S1. Crystal data and structural refinement for **TBT1**.

Identification code	**TBT1**	
CCDC deposition No.	2070566	
Empirical formula	$C_{18}H_{18}O_2S_4$	
Formula weight	394.61	
Temperature	169.99(10)	
Crystal system	triclinic	
Space group	P-1	
Unit cell dimensions	a = 4.7262(2) Å	α =105.021(3)°
	b = 7.9566(2) Å	β = 99.869(3)°
	c = 12.7539(4) Å	γ = 98.822(3)°
Volume	446.39(3) $Å^3$	
Z	1	
Density (calculated)	1.4678 g/m^3	
Absorption coefficient	4.956 mm^{-1}	
F(000)	207.8	
Crystal size	0.2 × 0.11 × 0.02 mm^3	
Radiation	Cu Kα (λ = 1.54184)	
2θ range for data collection	7.36 to 148.36	
Index ranges	$-5 \le h \le 5$, $-9 \le k \le 9$, $-15 \le l \le 15$	
Reflections collected	4861	
Independent reflections	1744 [R_{int} = 0.0325, R_{sigma} = 0.0229]	
Data / restraints / parameters	1744/1/130	
Goodness-of-fit on F^2	1.056	
Final *R* indices [I>=2σ (I)]	R_1 = 0.0744, wR_2 = 0.1754	
R indices (all data)	R_1 = 0.0759, wR_2 = 0.1766	
Extinction coefficient	n/a	
Largest diff. peak and hole	0.84/-1.03 e.$Å^{-3}$	

Table S2. Crystal data and structural refinement for **TBT2**.

Identification code	**TBT2**	
CCDC deposition No.	2070565	
Empirical formula	$C_{10}H_{11}S_2$	
Formula weight	195.31	
Temperature	170.00(10)	
Crystal system	monoclinic	
Space group	P21/c	
Unit cell dimensions	a = 12.4532(4) Å	α = 90°
	b = 5.9394(2) Å	β = 99.147(3)°
	c = 13.4612(4) Å	γ = 90°
Volume	982.99(5) $Å^3$	
Z	4	
Density (calculated)	1.320 g/m^3	
Absorption coefficient	4.414 mm^{-1}	
F(000)	412.0	
Crystal size	0.26 × 0.15 × 0.08 mm^3	
Radiation	CuKα (λ = 1.54184)	
2θ range for data collection	7.19 to 149.49	
Index ranges	-15 ≤ h ≤ 15, -7 ≤ k ≤ 7, -13 ≤ l ≤ 16	
Reflections collected	9988	
Independent reflections	1979 [R_{int} = 0.0334, R_{sigma} = 0.0130]	
Data / restraints / parameters	1979/0/111	
Goodness-of-fit on F^2	1.108	
Final *R* indices [I>=2σ (I)]	R_1 = 0.0487, wR_2 = 0.1261	
R indices (all data)	R_1 = 0.0490, wR_2 = 0.1263	
Extinction coefficient	n/a	
Largest diff. peak and hole	0.41/-0.47 e.$Å^{-3}$	

Table S3. Crystal data and structural refinement for **TBT3**.

Identification code	**TBT3**	
CCDC deposition No.	2103035	
Empirical formula	$C_{32}H_{46}O_2S_4$	
Formula weight	590.93	
Temperature	169.99(13)	
Crystal system	monoclinic	
Space group	C2/c	
Unit cell dimensions	a = 34.0453(16) Å	α = 90°
	b = 5.5520(2) Å	β = 106.469(4)°
	c = 17.6696(6) Å	γ = 90°
Volume	3202.9(2) $Å^3$	
Z	4	
Density (calculated)	1.225 g/m^3	
Absorption coefficient	2.922 mm^{-1}	
F(000)	1272.0	
Crystal size	0.26 × 0.15 × 0.08 mm^3	
Radiation	CuKα (λ = 1.54184)	
2θ range for data collection	5.414 to 150.372	
Index ranges	-42 ≤ h ≤ 41, -6 ≤ k ≤ 6, -21 ≤ l ≤ 17	
Reflections collected	9878	
Independent reflections	3120 [R_{int} = 0.0344, R_{sigma} = 0.0278]	
Data / restraints / parameters	3120/78/194	
Goodness-of-fit on F^2	1.059	
Final *R* indices [I>=2σ (I)]	R_1 = 0.0743, wR_2 = 0.2066	
R indices (all data)	R_1 = 0.0830, wR_2 = 0.2192	
Extinction coefficient	n/a	
Largest diff. peak and hole	1.17/-0.49 e.$Å^{-3}$	

3. ^{1}H and ^{13}C NMR spectra

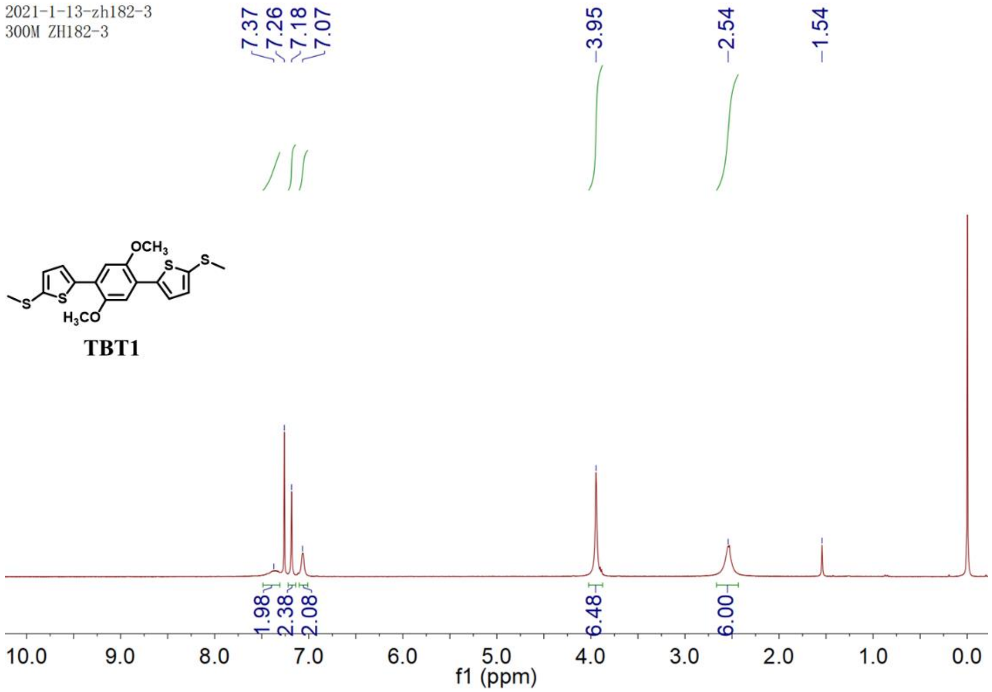

Figure S1. ^{1}H NMR spectrum of **TBT1** in $CDCl_3$.

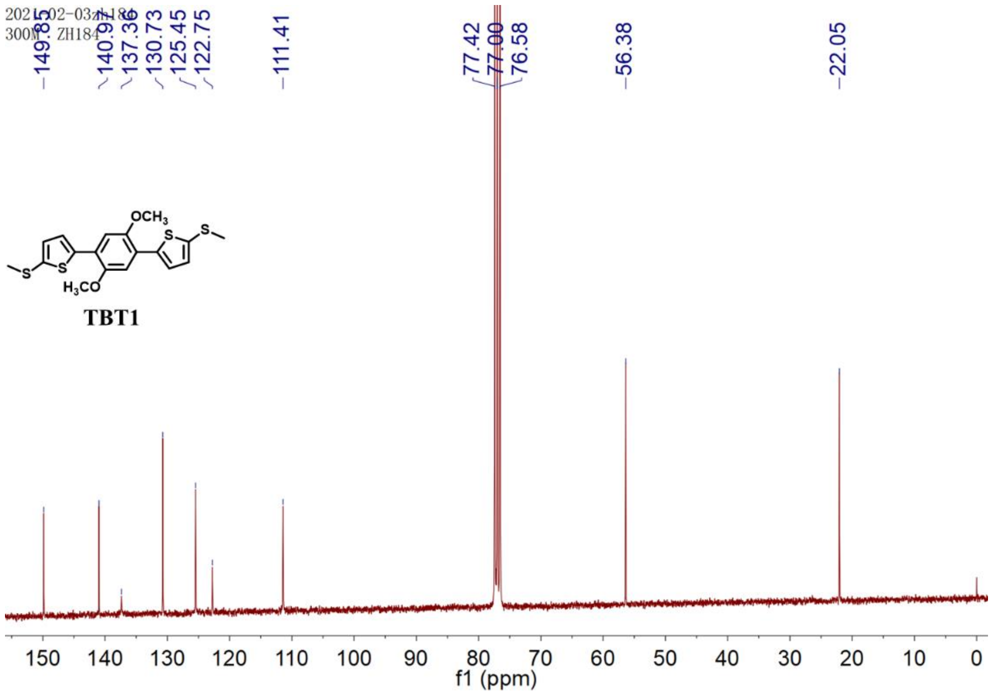

Figure S2. ^{13}C NMR spectrum of **TBT1** in $CDCl_3$.

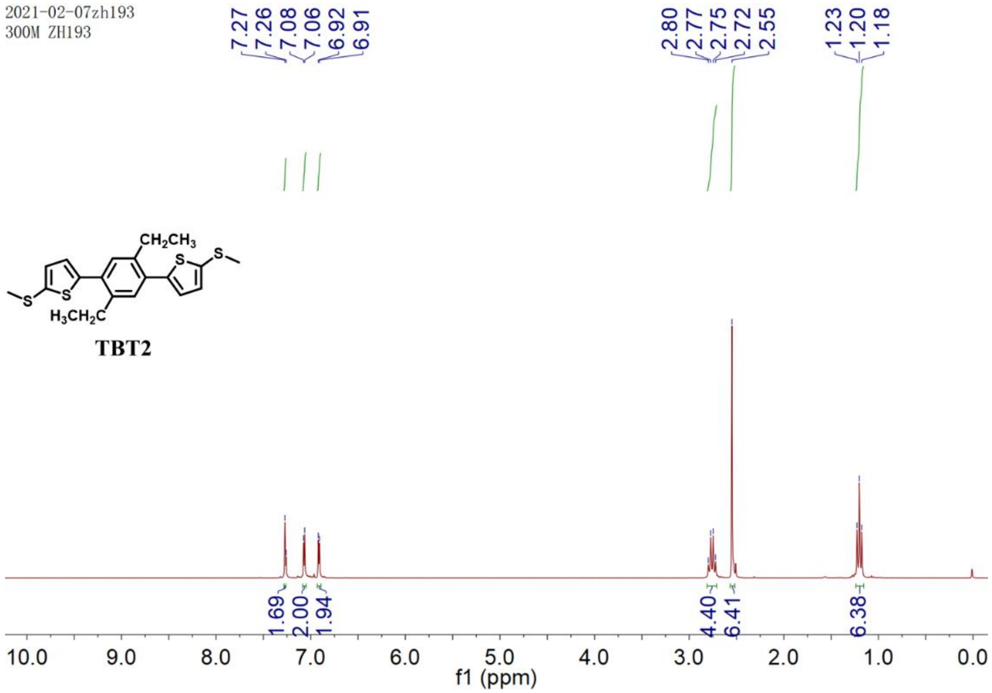

Figure S3. ^{1}H NMR spectrum of **TBT2** in $CDCl_{3.}$

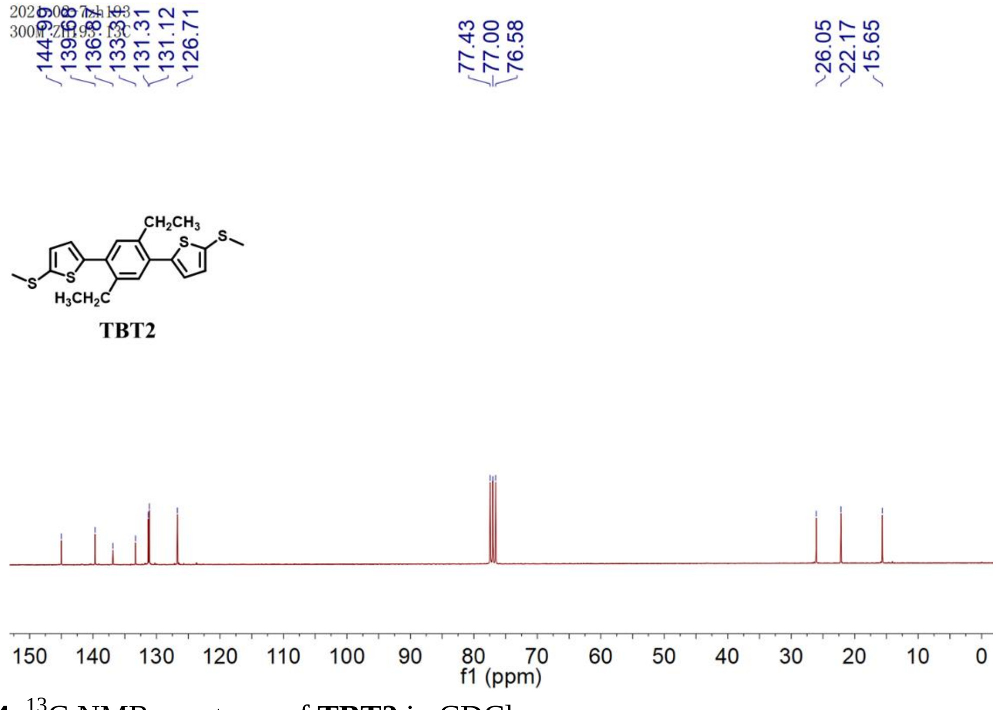

Figure S4. ^{13}C NMR spectrum of **TBT2** in $CDCl_{3.}$

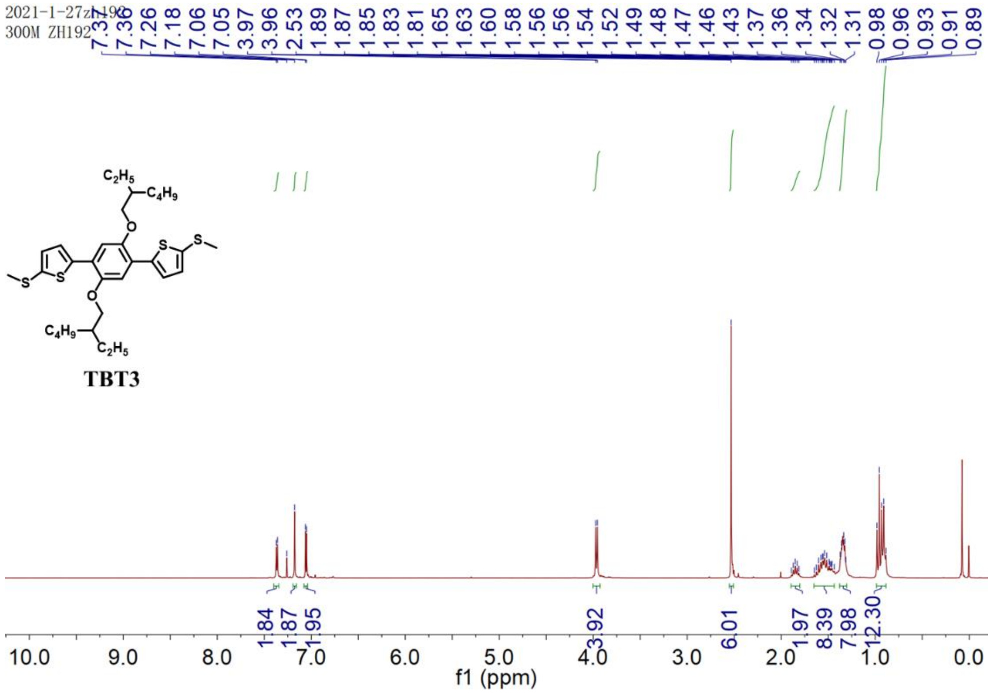

Figure S5. ^{1}H NMR spectrum of **TBT3** in $CDCl_{3.}$

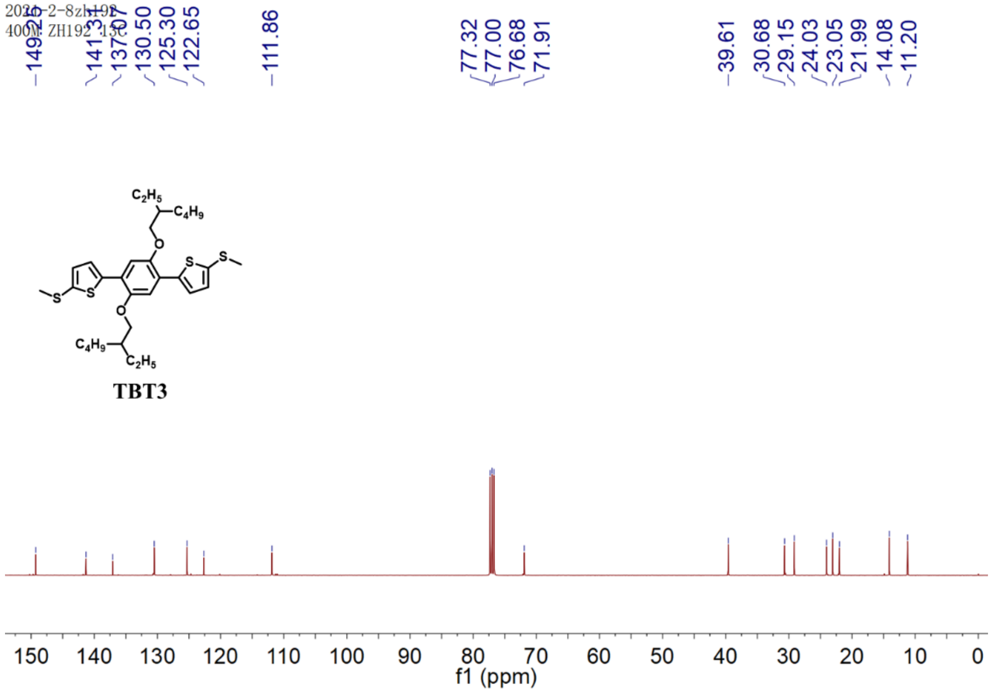

Figure S6. ^{13}C NMR spectrum of **TBT3** in $CDCl_{3.}$

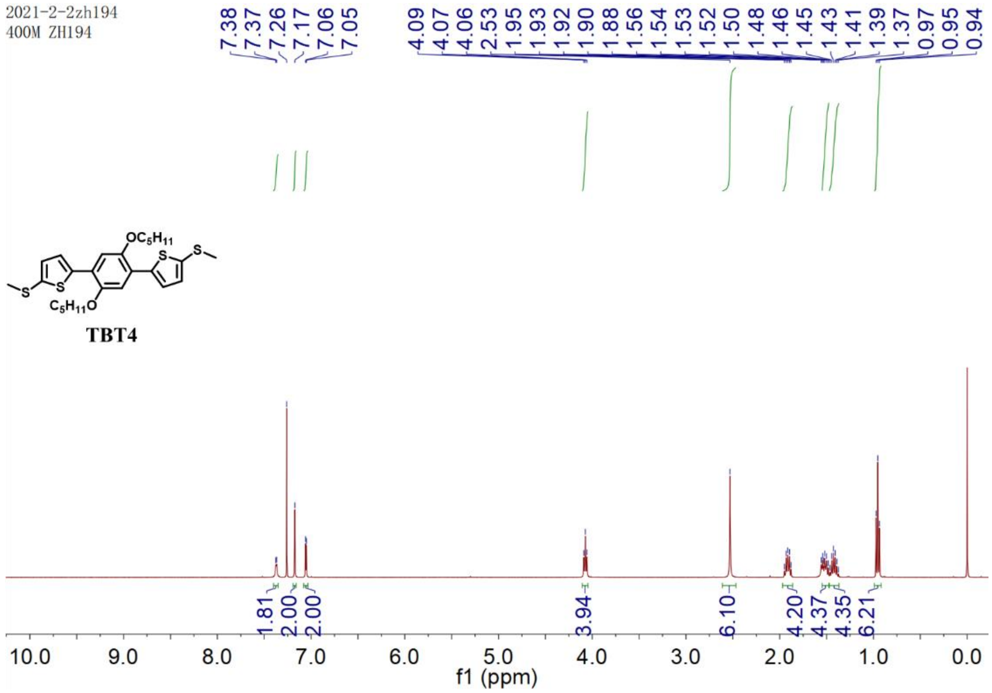

Figure S7. ^{1}H NMR spectrum of **TBT4** in $CDCl_3$.

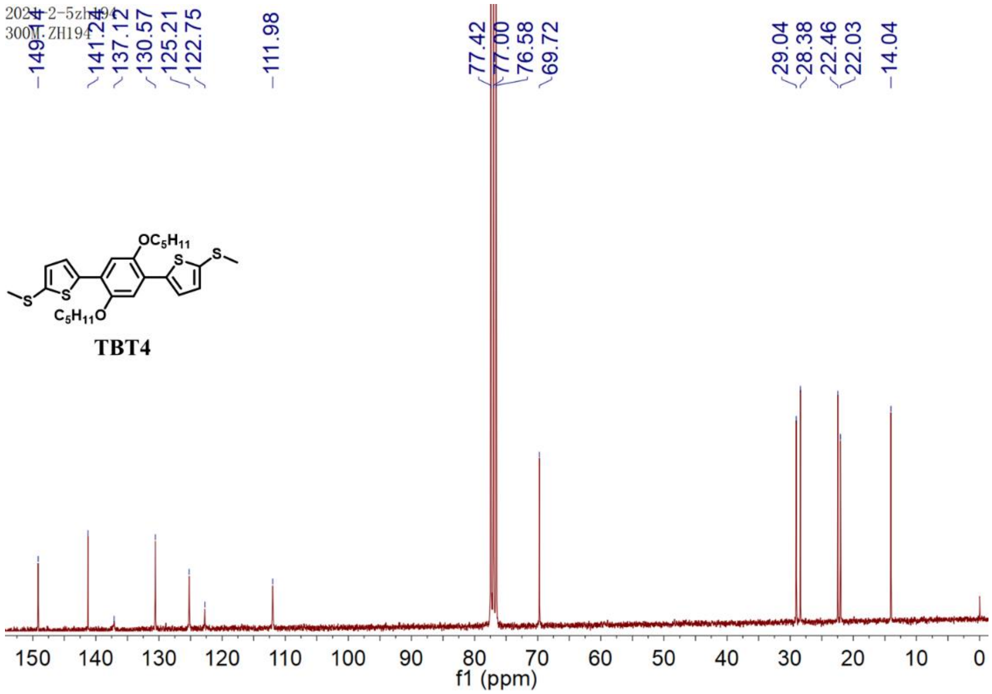

Figure S8. ^{13}C NMR spectrum of **TBT4** in $CDCl_3$.

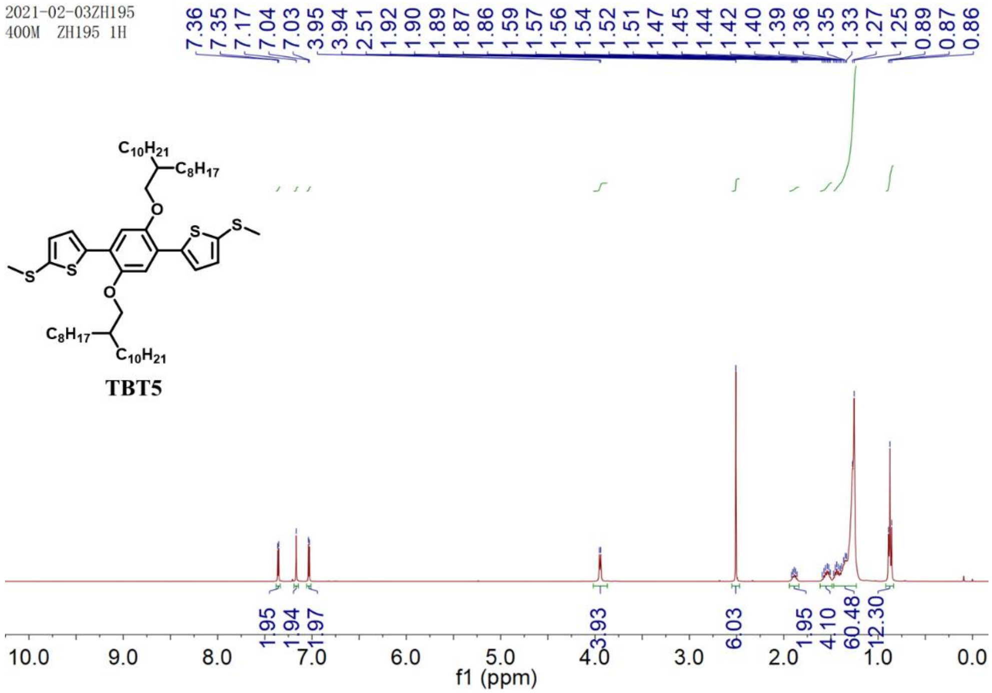

Figure S9. ^{1}H NMR spectrum of **TBT5** in $CDCl_3$.

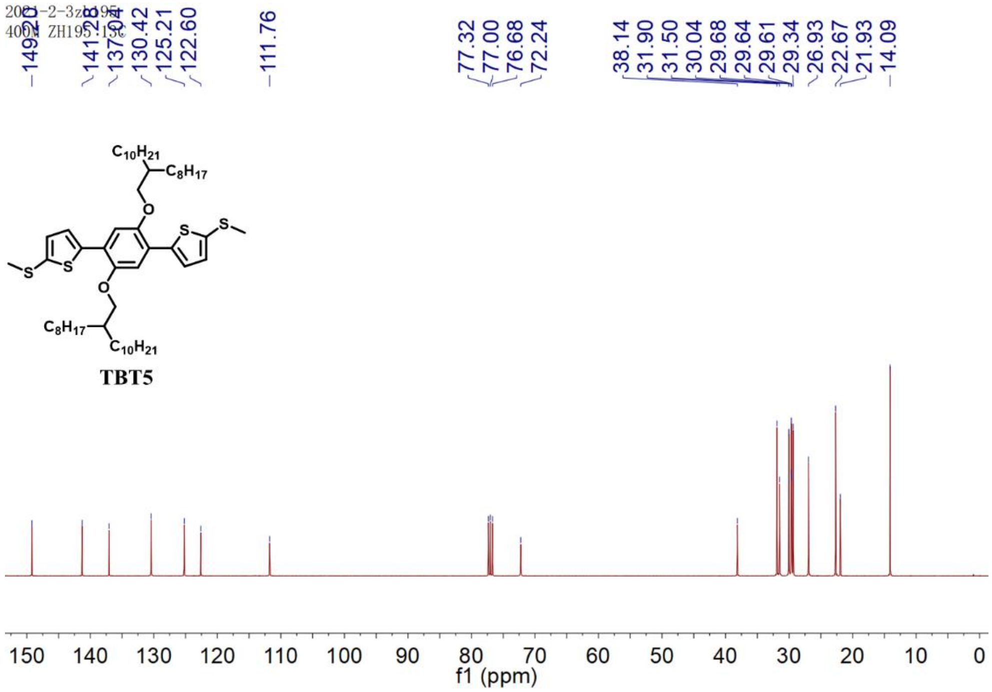

Figure S10. ^{13}C NMR spectrum of **TBT5** in $CDCl_3$.

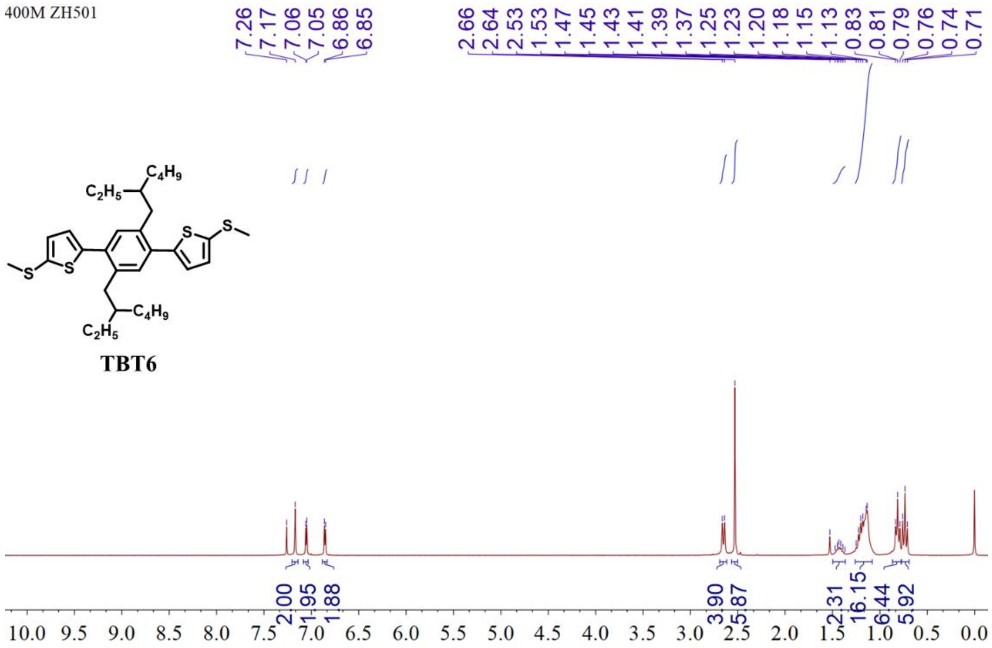

Figure S11. ^{1}H NMR spectrum of **TBT6** in $CDCl_{3.}$

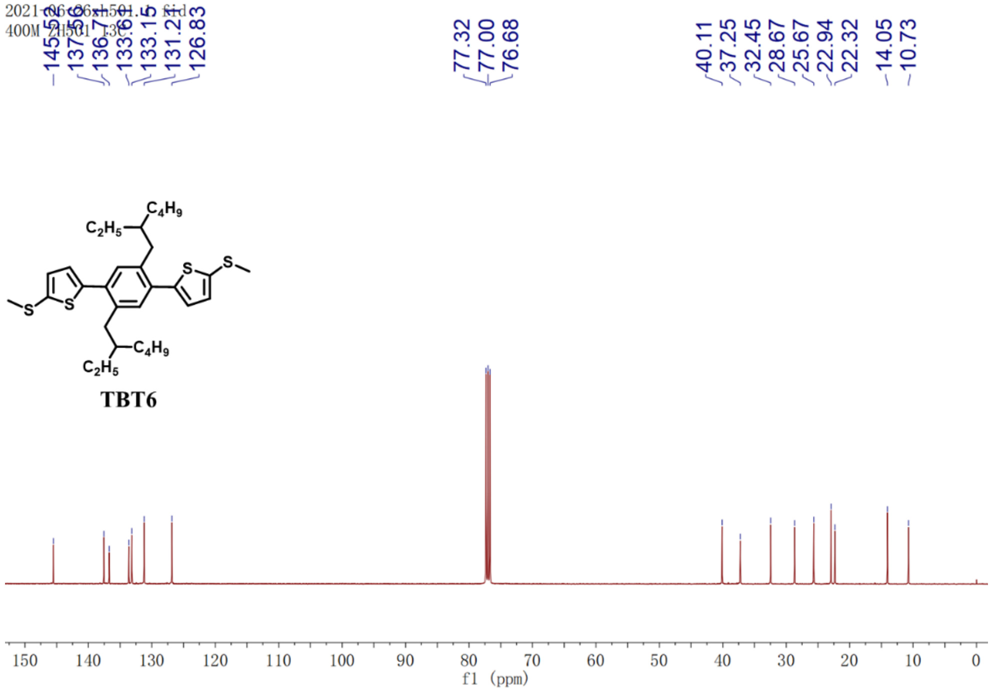

Figure S12. ^{13}C NMR spectrum of **TBT6** in $CDCl_{3.}$

4. Absorption spectra of TBT1, TBT2 and TBT3

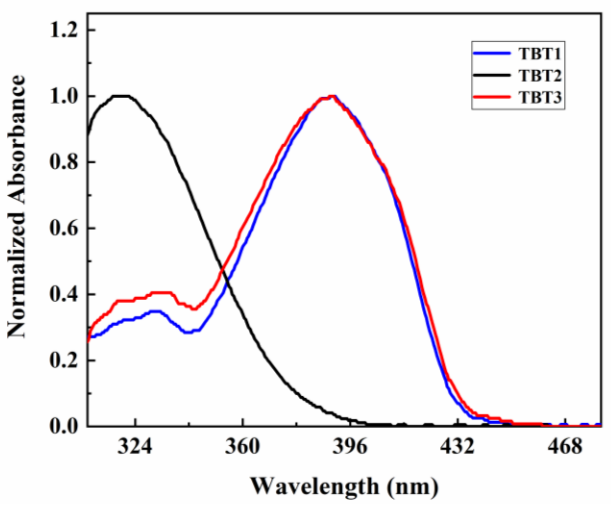

Figure S13. UV−vis absorption spectra for **TBT1**, **TBT2** and **TBT3** measured in 1,2,4-trichlorobenzene at a concentration of 10 μM.

5. Comparison of ^{1}H NMR spectra of TBT1, TBT2 and TBT3

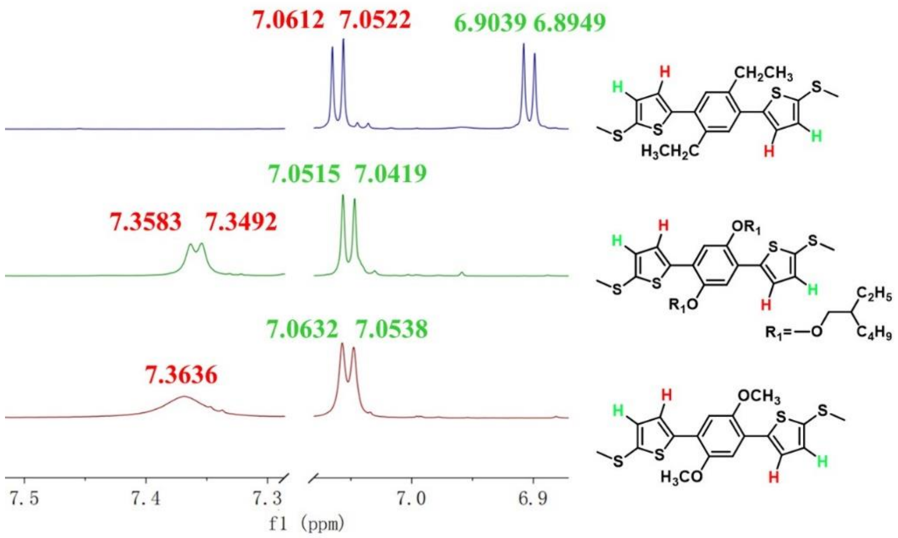

Figure S14. ^{1}H NMR spectra (298 K) of **TBT1**, **TBT2** and **TBT3** in $CDCl_3$.

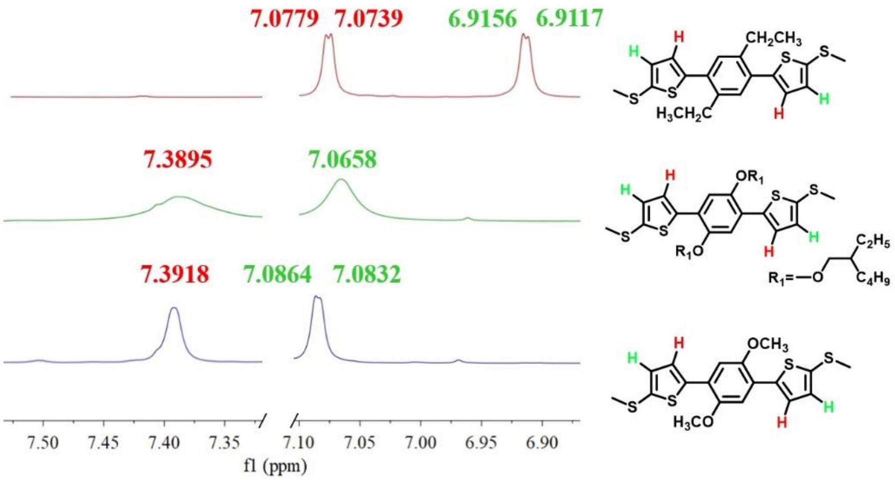

Figure S15. ^{1}H NMR spectra (233K) of **TBT1**, **TBT2** and **TBT3** in $CDCl_3$.

6. Theoretical modelling

DFT calculations: The relaxed geometry and Hamiltonian matrix elements of each molecular structure was obtained using the Fritz Haber Institute ab initio molecular simulation (FHI-aims) packages implemented by density functional theory (DFT). The FHI-aims method employs quantum-mechanical first principles to compute the total energy and derived quantities of molecules in its electronic ground state. The Perdew Burke-Ernzerhof parameterization (PBE) functional was considered for the exchange-correlation. The convergence for the self-consistency cycle based on energy derivatives (forces) is $1e^{-4}$ eV/Å.

Transport calculations: The ab initio transport simulations package (AITRANSS) was employed to calculate the electron transport characteristics for molecular junctions based on non-equilibrium Green's function formalism (NEGF). The molecular junction contains two Au electrode leads connected on both ends of targeted molecules. Each electrode was modeled by a pyramidal cluster of 60 Au atoms with the distance of 2.88 Å.

Calculations of the targeted molecules: We carried out the DFT calculations for molecules with -SMe anchors. The single Au atoms were attached to the S atoms at SMe anchors of the molecules to further optimize the geometries. To model the molecular configurations in electric field, we firstly applied electric field with 1V/nm to optimized the geometries as shown in Figure S15. After attaching single Au atoms to the two sides of the optimized molecules, the electric field with the direction along with two Au atoms was applied to relax the molecular geometries. The Au pyramids for calculating the transmission across the junctions after optimizing in the electric field.

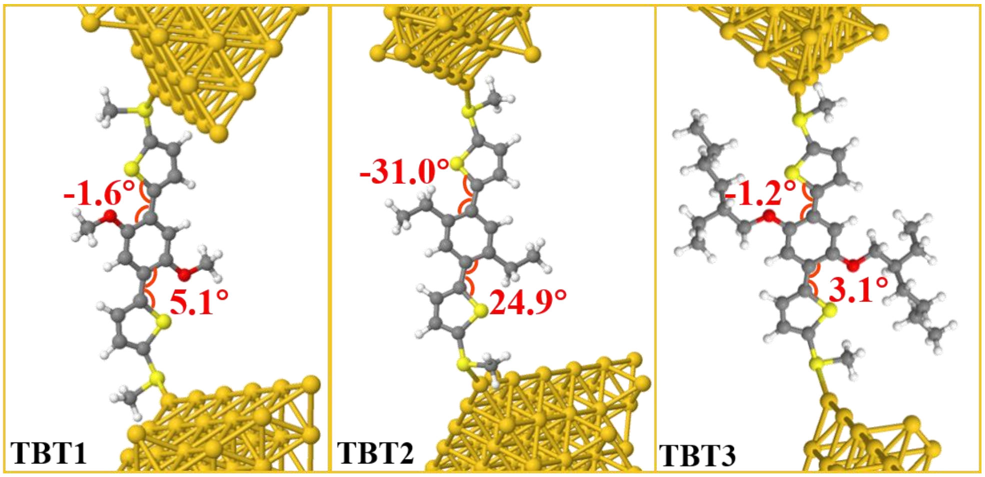

Figure S16. Theoretical calculations of the geometry configuration for **TBT1**, **TBT2** and **TBT3** under the electric field of 1 V/nm.

7. Data analysis of single-molecule conductance

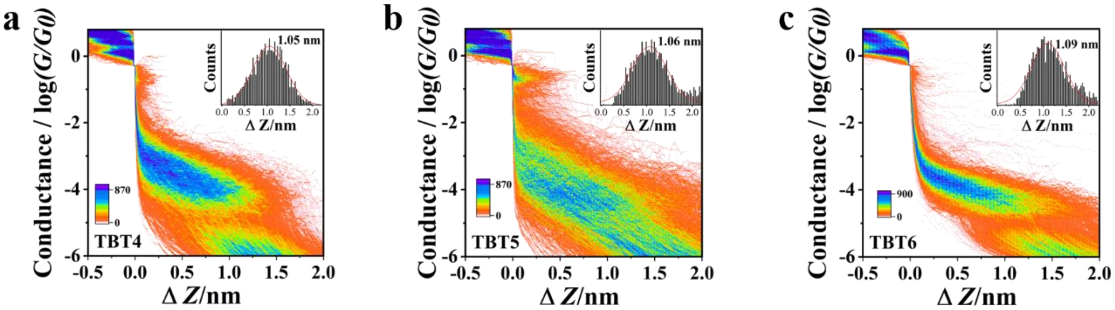

Figure S17. (a-c) 2D histogram of single-molecule conductance for **TBT4**, **TBT5** and **TBT6** under 0.1 V bias voltage. The distance distributions are shown in the insets.

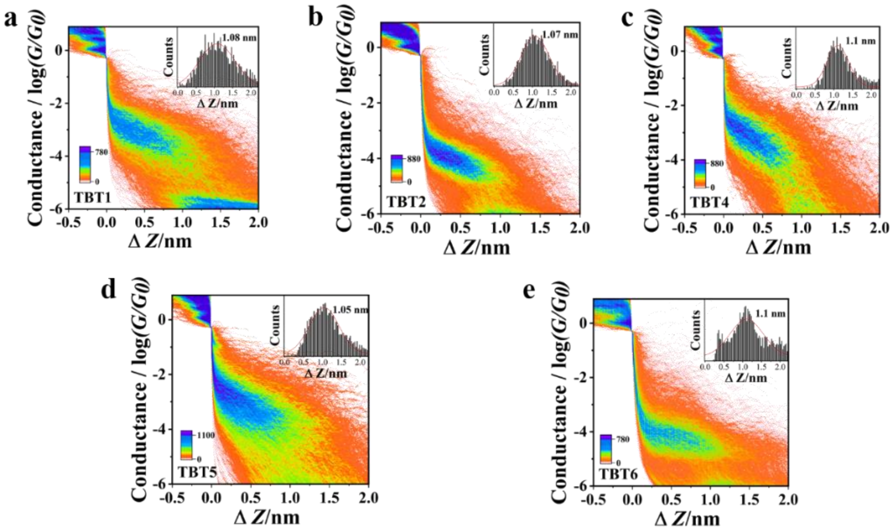

Figure S18. (a-e) 2D histogram of single-molecule conductance for **TBT1**, **TBT2**, **TBT4**, **TBT5** and **TBT6** under 0.5 V bias voltage. The distance distributions are shown in the insets.

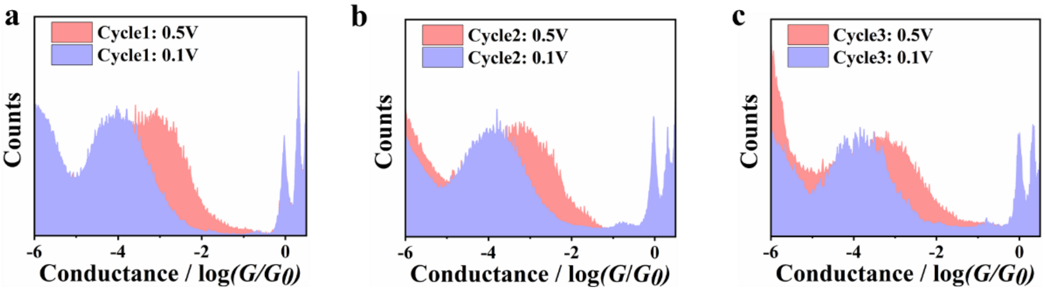

Figure S19 (a-c) 1D conductance histograms of reversible switching for **TBT3** under 0.1 V (blue) and 0.5 V (red) alternatively.

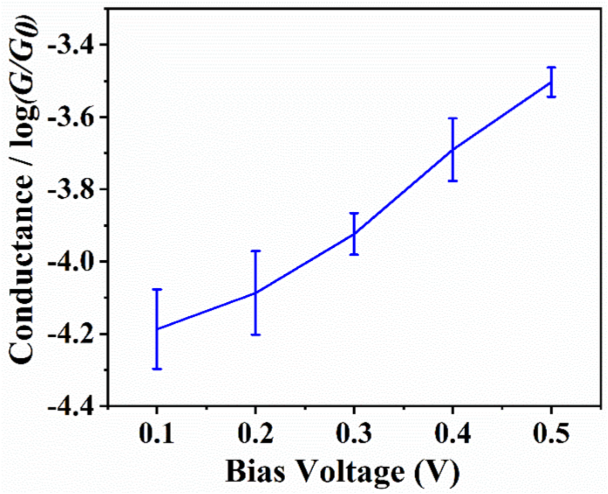

Figure S20 Molecular conductance of **TBT3** from 0.1 V to 0.5 V bias voltages.
